# Molecular Analysis of the Clavulanic Acid Regulatory Gene Isolated from an Iranian Strain of *Streptomyces Clavuligerus* , PTCC 1709

**Published:** 2011-09-23

**Authors:** Zohreh Hojati, Zahra Salehi, Majid Motovali-Bashi, Hasan Korbekandi, Saeed Jami

**Affiliations:** 1. Department of Biology, Faculty of Sciences, University of Isfahan, Isfahan, Iran; 2. Department of Genetics and Molecular Biology, Faculty of Medicine, Medical University of Isfahan, Isfahan, Iran

**Keywords:** S. clavuligerus, claR, Clavulanic acid

## Abstract

**Objective:**

The clavulanic acid regulatory gene (claR) is in the clavulanic acid biosynthetic gene cluster that encodes ClaR. This protein is a putative regulator of the late steps of clavulanic acid biosynthesis. The aim of this research is the molecular cloning of claR, isolated from the Iranian strain of *Streptomyces clavuligerus (S. clavuligerus)*.

**Materials and Methods:**

In this experimental study, two different strains of S. clavuligerus were used (PTCC 1705 and DSM 738), of which there is no claR sequence record for strain PTCC 1705 in all three main gene banks. The specific designed primers were subjected to a few base modifications for introduction of the recognition sites of BamHI and ClaI. The claR gene was amplified by polymerase chain reaction (PCR) using DNA isolated from S. clavuligerus PTCC 1705. Nested-PCR, restriction fragment length polymorphism (PCR-RFLP), and sequencing were used for molecular analysis of the claR gene. The confirmed claR was subjected to double digestion with BamHI and ClaI. The cut claR was ligated into a pBluescript (pBs) vector and transformed into *E. coli*.

**Results:**

The entire sequence of the isolated claR (Iranian strain) was identified. The presence of the recombinant vector in the transformed colonies was confirmed by the colony-PCR procedure. The correct structure of the recombinant vector, isolated from the transformed *E. coli*, was confirmed using gel electrophoresis, PCR, and double digestion with restriction enzymes.

**Conclusion:**

The constructed recombinant cassette, named pZSclaR, can be regarded as an appropriate tool for site directed mutagenesis and sub-cloning. At this time, claR has been cloned accompanied with its precisely selected promoter so it could be used in expression vectors. Hence the ClaR is known as a putative regulatory protein. The overproduced protein could also be used for other related investigations, such as a mobility shift assay.

## Introduction

*Streptomyces* species are mycelial, aerobic grampositive bacteria readily isolated from soil (1,2).*Streptomyces* are unique among prokaryotes due to their complicated morphological differentiation(3)These morphological changes are accompanied by a wide range of physiological events, including the production of secondary metabolites, many of which have potentially important biological activities. They include many useful antibiotics and other products, such as antitumor drugs and herbicides(4-8).*Streptomyces clavuligerus (S. clavuligerus)* produces the β-lactam antibiotic, cephamycin C and theβ -lactamase inhibitor, clavulanic acid(9-11).Clavulanic acid is a clinically significant inhibitor of β-lactamases, while the other clavam metabolites produced by *S. clavuligerus* demonstrate weak antibacterial and antifungal activities(1,9).Several other *Streptomyces* species have also been determined to be producers of clavulanic(12,13).The combined use of clavulanic acid and broad-spectrum β-lactam antibiotics such as amoxicillin are an important therapeutic tactic to combat the rapid increase in β-lactam resistance(14-17).The cluster of genes for clavulanic acid biosynthesis is located downstream from the pcbC gene of the cephamycin C cluster in *S. clavuligerus*(18,19).Most genes of the cephamycin and clavulanic acid clusters are known(20-24).All essential genes of the clavulanic acid pathway are within a 12kb* EcoRI* DNA fragment of the *S. clavuligerus* genome, because this fragment appears to confer production of clavulanic acid when introduced in *Streptomyces lividans*(25).Very little is known about the regulation of the genes of the clavulanic acid cluster. The transcriptional activators CcaR and ClaR are known to regulate the expression of clavulanic acid biosynthetic genes(26-28)The *ccaR* gene lies within the cephamycin biosynthetic gene cluster. This gene is a pathway-specific transcriptional regulator for cephamycin biosynthesis, as well as a controlling expression of the *claR* gene from the clavulanic acid gene cluster(21,29-31)Another regulatory gene, claR, is located immediately downstream from *orf-7* in the clavulanic acid cluster and encodes a 431 amino acid protein(31,32).The regulatory nature of the ClaR protein has been deduced from the presence of one helix turn helix (HTH) motif and flanking sequences which show significant similarity to LysR transcriptional regulators(33). Finally, the absence of *orf-7, orf-9* and *orf-10* transcripts in a *claR* mutant blocked in clavulanic acid production confirmed the regulatory role of ClaR(32-34). 

 To increase the amount of clavulanic acid produced by *S. clavuligerus*, different tactics have been employed by researchers. Enhancement of clavulanic acid production was seen in* S. clavuligerus* in the presence of peanut *(Arachis hypogaea)* seed flour and its fractions(35). Random mutagenesis was performed on *S. clavuligerus*. The new mutated strains were able to produce the elevated level of clavulanic acid(36).

Since clavulanic acid is produced industrially by fermentation using *S. clavuligerus*, the regulation of clavulanic acid biosynthesis is a point of great interest. It has been shown that the cloning of the claR gene in the *S. clavuligerus* resulted in a threefold increase in clavulanic acid production(31).In our previous work, an isolated claR gene was ligated to a *Streptomyces* specific vector (pMA::hyg). The cloned claR genes had been isolated from two standard strains of *Streptomyces*.

 In this work, a new recombinant construct that carries the *claR* regulatory gene is presented. This vector not only transfers the claR gene isolated from one Iranian strain of *S. clavuligerus*, but also contains an inducible promoter.

## Materials and Methods

### Bacterial strains

*S. clavuligerus* DSM 41826 (DSM, Germany) and *S. clavuligerus* PTCC 1705 (Iranian Scientific and Industrial Research Organization, Iran) were used in this study.* Escherichia coli (E. coli)* XL1-Blue was also used in this study. The *Streptomyces* strains were grown in defined conditions as described previously (37). A suspension of *Streptomyces* spores was prepared in 20% (v/v) glycerol and stored at -20℃ (38). Cultures for the isolation of chromosomal DNA were prepared by inoculating 100 ml of yeast extract medium (YEM) with 100 µl of spore suspension. The YEM medium was prepared as described previously (37). Luriabertani (LB) agar medium (that contained per liter: 10 g of trypton, 5 g of bacto-yeast extract, 10 g of NaCl and 17 g of agar; pH= 7.5) supplemented with Ampicillin (100 µg/ml), whenever required, was used for the propagation of* E. coli* at 37℃. The bacterial pellet was stored in 20% glycerol at -20℃.

### Vector

The pBs SK reproduced from Stratagene Catalogue was used as the vector in this study.

### Primers

**Fig1:**
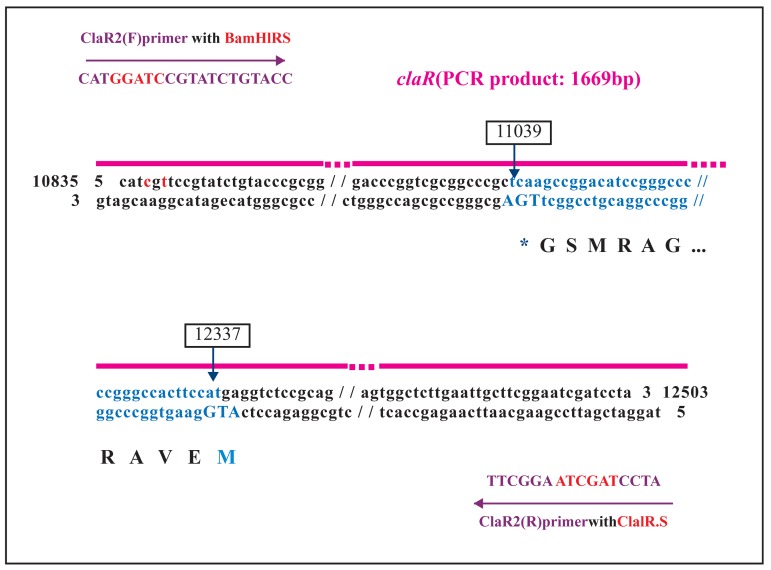
Nucleotide sequence of the claR gene. The main primers have been subjected to a few nucleotide modifications as shown here after transplantation and orchidopexy.

OLIGO® version 5.0 software (39) was used for designing all primers. The entire coding region of the gene was considered for primer selection. Accession number AJ000671.1, GI:2764535 (or U87786.2, GI:9280818) was used for obtaining the *claR* sequence. These accession numbers are based on *S. clavuligerus* ATCC 2706. This strain is the same as* S. clavuligerus* DSM 738, as mentioned in the NCBI. One set of primers (claR1) was designed for nested PCR (F: 5'GCC TGG AGC AGA TGG AG 3'and R: 5'AGG TGC TGT CGC TGG TCT 3'). Two primers (claR2) were designed for isolation of the *claR* gene from genomic DNA of *S. clavuligerus* (F: 5'CAT GGA TCC GTA TCT GTA CC 3' and R: 5'TAG GAT CGA TTC CGA AGC 3'). These primers were subjected to modification at each 5' end in order to have two recognition sites for *Bam*HI and *Cla*I (Fig 1).

### Separation of total genomic DNA from Streptomyces

Total genomic DNA was isolated from the liquid culture of *Streptomyces* using the High Pure PCR Template Preparation Kit (Roche; Cat. No.1 796 828). The amount of DNA was quantified by gel electrophoresis and spectrophotometric analysis.

### Polymerase chain reaction (PCR )

 The reaction mixture for PCR amplification was prepared as follows: forward primer, 20 pM; reverse primer, 20 pM; dimethyl sulfoxide (DMSO), 4 µl; 10×PCR buffer without MgSO4 (200 mM Tris-HCl, 100 mM (NH_4_)_2_SO_4_, 100 mM KCl, 1% (v/v) Triton X-100, 1 mg/ml bovine serum albumin (BSA)), 5 µl; MgSO_4_, 3 µl; deoxynucleoside triphosphates (dNTPs), 2 µl (10 mM each dNTP); and H_2_O, up to 50 µl. A total of 100 ng of chromosomal DNA was added as the template DNA. The PCR reactions were then carried out using 0.3 µl (2.5 U/µl) of *Pfu* polymerase enzyme. The amplification steps for the main PCR were as follows: hot start at 95℃ for 5 minutes; 33 cycles of denaturation at 94℃ for 1 minute, annealing at 60℃ for 1 minute, primer extension at 72℃ for 4 minutes, and a final extension at 72℃ for 15 minutes. These conditions were set up for the modified primers. The amplification procedure was slightly different for the nested primers. The PCR was carried out normally for 30-35 cycles. The products were visualized by a standard electrophoresis procedure using 0.7% (W/V) agarose gels.

### Restriction endonuclease (RE) digestion

Two sets of primers were designed not only to amplify the claR region, but also to integrate one unique recognition site (*Bam*HI and *Cla*I) in each end of the amplified fragments. Digestion was performed following the recommendations of the manufacturer (Fermentas, Germany). Required amounts of DNA samples (0.2-5 µg) were generally digested with 5-10 units of restriction enzymes (*Bam*HI and *Cla*I) in a 10-20 µl final volume of restriction buffer (10× buffer) for about 1-3 hours in a water bath at the recommended temperature (normally 37℃). A sample was run on an agarose gel after incubation with each enzyme, which ensured that the digestion was done completely (40).

### DNA ligation

 DNA ligation was performed using one unit of T4 DNA ligase (Fermentas, Germany) in the presence of 1× ligation buffer. The 3:1 molar ratio of insert to vector was used in order to optimize transformation. Incubation was done at 16℃ overnight (40). The products of the ligase reaction (a 20 ng aliquot from the completed ligase mixture) were analyzed by electrophoresis on a 2 × 50 ×75 mm agarose gel (mini gel).

### Transformation of E. coli

For making competent cells from E. coli, the calcium chloride method was used (40). An aliquot (200 µl) of frozen competent cells were slowly thawed on ice for about 30 minutes. Cells were gently mixed with DNA and incubated on ice for 30 minutes. The cells were then heat shocked at 42℃ for 90 seconds. They were added to 2 ml LB (without antibiotic) and incubated at 37℃ for one hour in a shaking incubator. A total of 100 µl of transformed cells were spread on the surface of the LB plate that contained an antibiotic. The plates were allowed to dry before overnight incubation at 37℃ (40).

###  Extraction of plasmid DNA from E. coli

 E. coli DNA was isolated according to the method described by Holmes and Quigley (40). The overnight LB culture of *E. coli* was harvested by centrifugation (13K rpm, 30 seconds). The pellet was re-suspended in 350 µl of STET [0.3 M NaCl, 10 mM Tris-HCl (pH= 8.0), 1mM EDTA (pH= 8.0), 0.5% Triton X-100] buffer, and subsequently 25 µl of freshly prepared lysozyme solution (10 mg/ml lysozyme in 10 mM TrisCl) was added. The tube that contained bacterial lysate was placed in a boiling water bath for 40 seconds before centrifugation at room temperature (12K rpm, 10 minutes). The pellet of bacterial cell debris was removed using a sterile toothpick. Plasmid DNA was precipitated with cold sodium acetate and isopropanol, washed with 70% ethanol, and re-dissolved in 50 µl of TE containing 10 g/ml RNase (40).

### DNA sequencing 

DNA sequencing was carried out using the Applied Biosystem (ABI) system (Bioneer, Italy).

## Results

### Isolation and molecular analysis of claR gene

**Fig 2:**
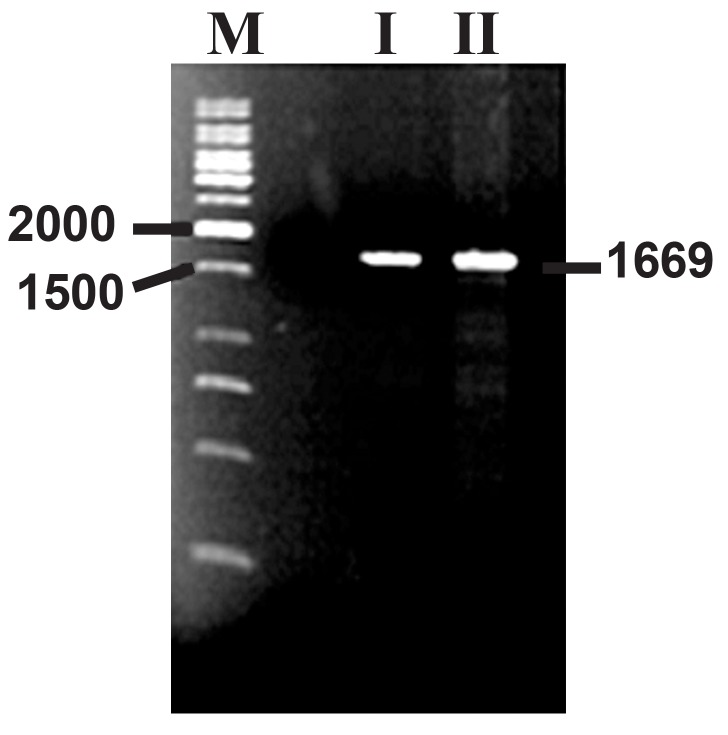
Amplification of claR with its native promoterfrom S. clavuligerus PTCC 1705 by PCR. I. S. clavuligerus total genomic DNA (isolated either from DSM738 or PTCC 1705) was used in the PCR reaction. The numbers are in base pair (bp), II. PTCC1705, III. DSM738, M. Marker; GeneRulerTM 1kb DNA ladder.

 Total DNA was isolated from *Streptomyces* and subjected to gel electrophoresis to analyze the concentration and purity. The pure, isolated total DNA was used for PCR reactions. Two different sets of primers were used. The claR gene was successfully amplified by using the claR2 primer set(Fig 2).

The isolated fragment had to be studied in more detail to further compare it with the original *cla*R of *S. clavuligerus* DSM738. Two different strategies were conducted to not only confirm the amplified fragment as claR gene, but to also compare it with the claR gene sequence from *S. clavuligerus* DSM738. Initially nested PCR, using the claR1 primer set, confirmed the existence of claR gene (data not shown).

**Fig 3:**
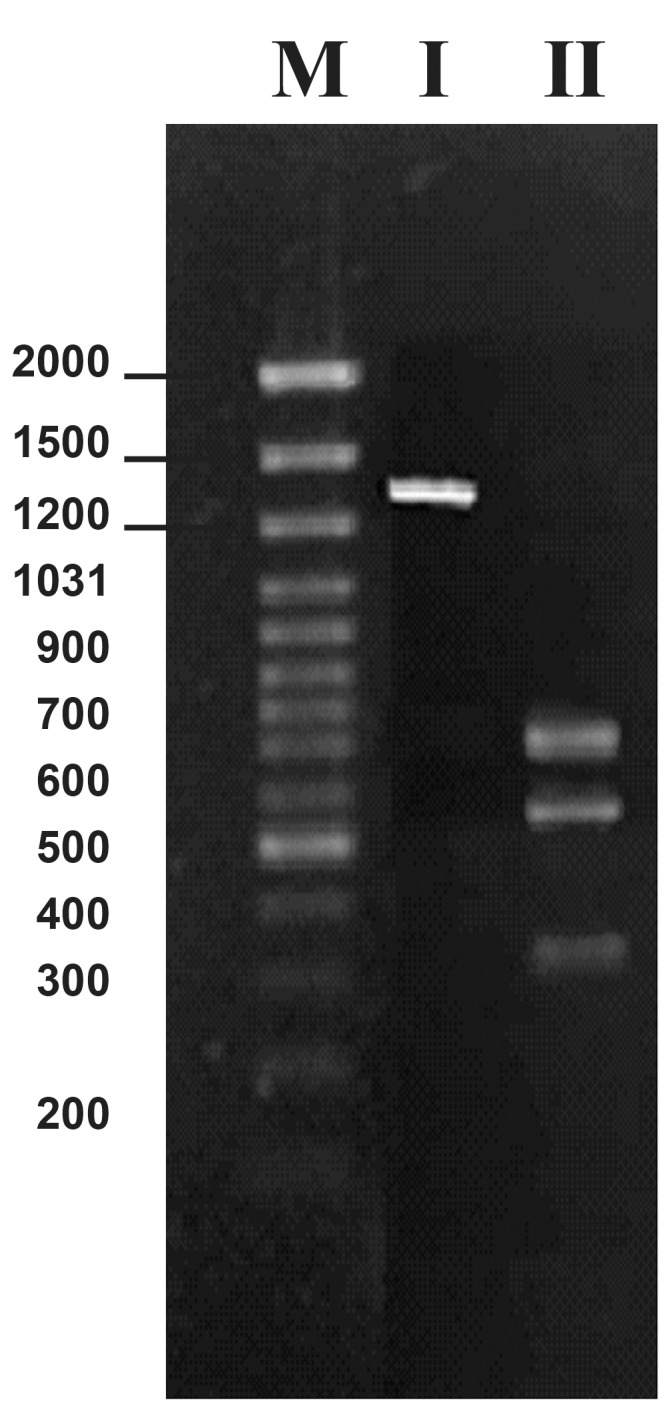
Molecular study of the claR, using restriction digestion analysis. PCR amplified claR was cut with different restriction enzymes. The results are visualized by gel electrophoresis (2%). The numbers are in bp. I. Cut with XbaI: This enzyme does not cut the claR fragment, II. Cut with SalI: This enzyme cuts the claR at two sites 740 and 1331 (leaving three fragments, 740 bp, 591 bp and 338 bp), M. Marker; GeneRulerTM 100 bp DNA Ladder Plus.

 On the other hand, the results of the nested PCR have confirmed the approximate similarity between these two genes (claR isolated from Iranian *S. clavuligerus* and *S. clavuligerus* DSM738). This conclusion was achieved because the *cla*R sequence of *S. clavuligerus* DSM738 had been used to design the primers. RFLP- PCR was then carried out using the *Sal*I restriction enzyme. *Sal*I cuts the claR gene at 740 and 1331 bp, producing three fragments, 740, 591 and 338 bp. The resultant fragments confirmed the correct structure for the isolated *cla*R gene(Fig 3).

**Fig 4:**
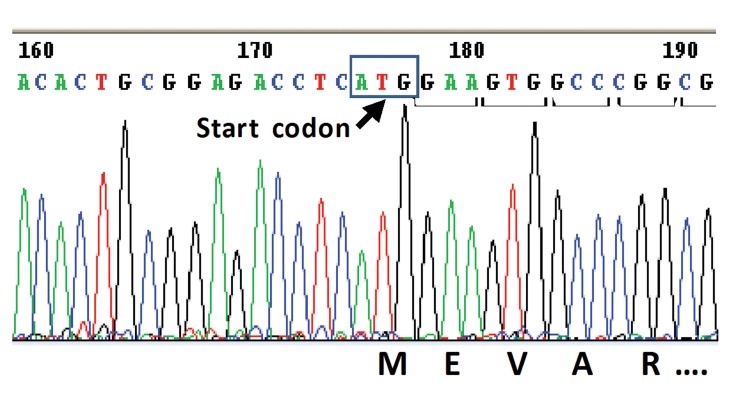
Structural analysis of the cloned claR, isolated from S. clavuligerus PTCC 1705. The start codon (ATG) of the claR gene has been shown here along with a few initial sequences related to amino acids E, V, A, and R. Not all the sequences have been shown

 Sequencing analysis of the *cla*R gene revealed that the *cla*R gene was amplified and sub-cloned, free from any mutation that was also essential for the correct expression of the gene. Bioinformatics analysis determined the complete similarity between the isolated *cla*R of the Iranian strain* S. clavuligerus* and *S. clavuligerus* 738(Fig 4).

The sequence of the claR gene from *S. clavuligerus* PTCC 1705 was determined for the first time in this study and will be submitted to the DDBJ/EMBL/ GenBank databases in the near future.

### Cloning of the claR gene

*E. coli* XL1-Blue was transformed with pBS plasmid. The pBs plasmid was then isolated from the transformed *E. coli* and subjected to double digestion (with *Bam*HI and *Cla*I), gel electrophoresis, and gel purification. The PCR amplified fragment was also double digested with *Bam*HI and *Cla*I, and the resultant cut fragment was purified by gel electrophoresis. A ligation mixture was set up using the double digested vector and the *cla*R gene. *E. coli* XL1-Blue competent cells were transformed using 10 µl of the ligation mixture. About 20 colonies were observed on each plate, which was inoculated with 100 µl of the transformed cells of *E. coli* XL1-Blue.

**Fig5:**
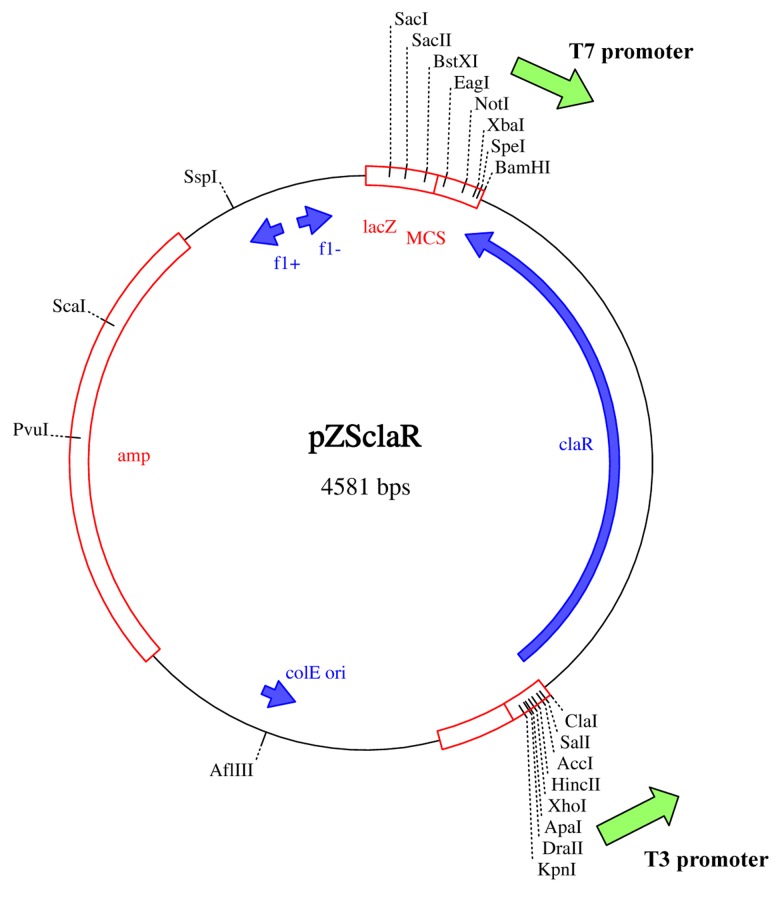
A physical map of the vector pZSclaR, 4581 bp. This plasmid map was drawn using computer software Clone Manager 6

**Fig 6:**
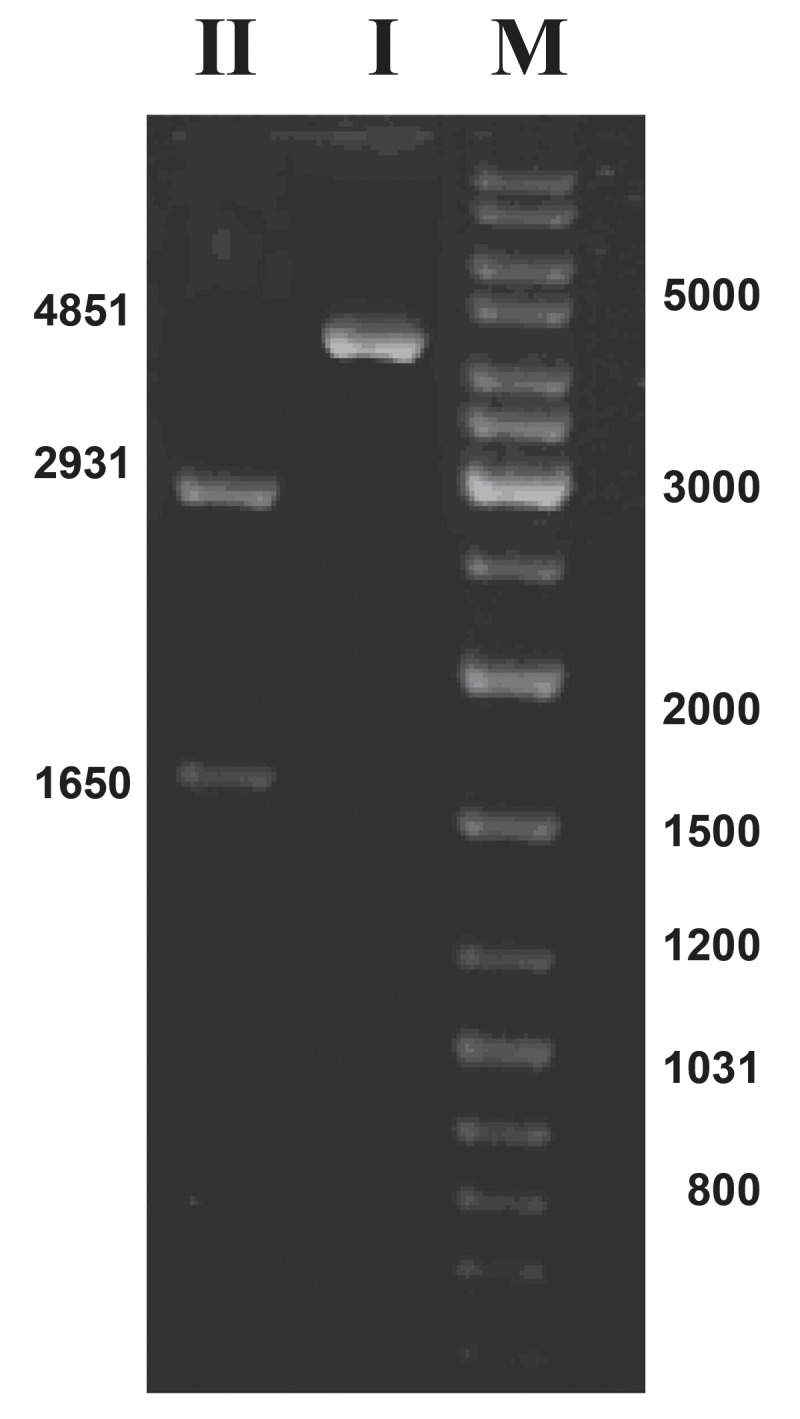
Structural confirmation of the pZSclaR using restriction digestion analysis. The pZSclaR plasmid was subjected to double digestion with BamHI and ClaI. The cut fragments were subjected to gel electrophoresis (0.7% ). Numbers are in bp. I. pZSclaR plasmid (uncut: 4581 bp), II. pZSclaR plasmid cut with BamHI and ClaI, M. Marker Gene Ruler™ 1kb DNA Ladder.

Therefore, the recombinant plasmid was isolated from a transformed colony. Molecular studies were then conducted on a 4581 bp new construct named *pZScla*R(Fig 5). The isolated plasmid was subjected to gel electrophoresis for initial confirmation of the size of the constructed vector (Fig 6) .

*pZSclaR* was then cut with *Bam*HI and *Cla*I for further confirmation of its structure and the resultant fragments were separated and visualized by gel electrophoresis.

 These two enzymes cut the *pZScla*R plasmid (4581) and separated the *cla*R gene (1650 bp) from the original vector (pBs; 2931 bp). *pZScla*R was then used as the PCR template in a PCR reaction containing nested primers that could confirm the existence of the *cla*R gene. Therefore the correct recombinant plasmid did exist in the recombinant strain of *E. coli*.

## Discussion

The overall aim of this work was to expand our knowledge of the regulation of antibiotic production in *Streptomyces* (the producer of two thirds of all known microbial antibiotics). Genetic engineering of the clavulanic acid producing strains could be done afterwards, in order to increase the capacity of clavulanic acid production in *S. clavuligerus*. It has been reported that *cda*R, the regulatory gene for the production of a calcium dependent antibiotic (CDA), positively regulates its own transcription. As a result, introducing extra copies of cdaR into different strains of *Streptomyces coelicolor* MT1110, S. coelicolor 2377 and *Streptomyces lividans* has led to overproduction of this antibiotic (41). Designing novel antibiotics, on the other hand, is greatly dependent on the structural analysis of the gene cluster for each antibiotic. Clavulanic acid is a multi-billion-dollar per annum product useful for its β-lactamase inhibitory activity. While the biosynthesis of clavulanic acid has been the subject of intense investigation in recent years, the details of its production and regulation are still not completely worked out.

Amplification of the ccaR gene, a regulatory gene in the cephamycin gene cluster, resulted in an almost threefold increase in the production of both cephamycin and clavulanic acid in *S. clavuligerus* (20). The formation of clavulanic acid is controlled by a LysR-type regulatory protein encoded by the claR gene. The *cla*R gene was then chosen because this is a putative regulatory gene in the production pathway of clavulanic acid (33). The *cla*R gene, which is located downstream from the gene encoding clavaminate synthase in the clavulanic acid biosynthesis gene cluster, is involved in regulation of the late steps in clavulanic acid biosynthesis (32-34). . Amplification of the claR gene using multi-copy plasmids and under its own promoter in *S. clavuligerus* results in a three-fold increase in clavulanic acid production(31).

We precisely amplified the coding sequence of *cla*R accompanied with its promoter by using a specifically designed primer and an error proof PCR. In this case, only the promoter sequence of the gene comes with the *cla*R. Since the distance between the vector born promoter and the claR transcription start point is not too great, the expression of the cloned gene could also be started by two individual promoters that exist in the vector. Therefore, the usage of three promoters (one native claR gene and two vector-born) leads to an elevated level of *cla*R gene expression. Prior to this study and in contrast to other regulatory genes in *S. clavuligerus*, *cla*R has not been isolated by PCR, but has been previously cloned via restriction enzyme digestion (31). For the first time, in the present study, the claR gene was isolated from an Iranian strain of *S. clavuligerus* PTCC 1705. The PCR isolated *cla*R was initially compared with the *cla*R sequence of *S. clavuligerus* DSM 738, using nested PCR and restriction digestion analysis. The *cla*R isolated from the *S. clavuligerus* PTCC 1705 was finally sequenced. Thus the entire sequence of *S. clavuligerus* claR was determined. The sequencing data was subjected to bioinformatics analysis for further comparison with the claR sequence of *S. clavuligerus* 738 and other species. Complete similarity was found between the sequence of claR isolated from PTCC 1705 and*S. clavuligerus* DSM 738.

 In our previous work, *cla*R was ligated into a *Streptomyces* specific vector, pMA::hyg (39). However that vector does not have any inducible promoter. On the other hand, pMA::hyg does not contain any other cut sites for other restriction enzymes (39), so the subcloning of *cla*R was practically impossible. To overcome these problems, new primers were designed with new incorporated cut sites for *Bam*HI and *Cla*I. The amplified *cla*R was then cloned in *E.coli* by using a newly constructed vector called *pZScla*R (Fig 4). This unique vector contains a greatly expanded multiple cloning site (MCS), which makes it suitable for different purposes of gene cloning. Furthermore, this new construct is expression and inducible. In the same way, increasing the copy number of certain clavulanic acid-specific biosynthetic genes, by the introduction of multiple copy expression plasmids, resulted in positive effects on the production of clavulanic acid (42).

## Conclusion

Characterization of isolated *cla*R from an Iranian strain of *S. clavuligerus* PTCC 1705 was carried out using molecular studies. This gene was cloned in *E. coli* via a multiple copy expression vector. The constructed recombinant cassette (*pZScla*R) may also be utilized as an appropriate tool for site directed mutagenesis and sub-cloning. The *Cla*R is recognized as a putative regulatory protein, so the overproduced protein could also be used for other related investigations, such as an enzyme assay and a mobility shift assay. The *cla*R gene could also be expressed in *Streptomyces* by sub-cloning it into different varieties of *Streptomyces* specific expression vectors. 
